# The Penguin Dictionary of Curious and Interesting Words

**Published:** 1987-08

**Authors:** J. D. Davies


### Book Review

**THE PENGUIN DICTIONARY OF CURIOUS AND INTERESTING WORDS**
**G. S. Saussy III**
**Penguin Books, Harmondsworth**
**ISBN 0 14 008520 3. Price £3.95**
**pp. 277+xii, paperback, 1986**

Our youngest daughter, now 18, was determined to talk even at the age of six months. She then shot out a string of inchoate expression delivered at high speed. Alas all, except the determination, was incomprehensible to the rest of the family. Mr Saussy, onetime wine salesman, steelyard supervisor and environmental planner seems to have the same urge to understand and to delight in the incomprehensible. American English is a cauldron of new expression. More cautiously, English English is changing too.

The source of this dictionary is both modern and older, now classical, English. The full range from Shakespeare to Anthony Burgess and American contemporaries is included.

The words are the essence of the book, and are great fun. They can delight, and do tempt. How set in our ways we can all get in medicine. Merely for an example, how about the word CASEATION? Here's a delightfully unexpected non-medical use of the *'noun'*, meaning the process of making cheese: "...a stinking anthology of Italian caseation..." (*vide* Anthony Burgess, 1980). Would that even professional tissue pathologists could understand that this is all the term caseation means. Being of cheesy character is not synonymous with tuberculosis. Being stinking and necrotic does not necessarily mean tuberculous.

Next, how about..., well really there is too much on offer. You only need to pick up the book itself.

All this Saussy-III-style American and English language is a strong counterblast to the good sense of our own eminent Messrs Fowler, Gowers and Fraser who recommend using only the plain and ordinary word in everyday English for official communication. Their publishers would be shocked by this new Penguin carnival of unusual usage.

How should we react?

I must admit, just for once, it would be refreshing to read an enthusiastic medical paper written in Saussy III language. Who knows, we might all read beyond the title and its abstract.

Even if most formal medical journals are less than keen on real English, the paperback price should not deter you from buying this gem. Messrs Penguin and Mr Saussy need our congratulations. I do hope that you too will enjoy this amazing paperback book.

J. D. Davies

